# Thinking together: What makes Communities of Practice work?

**DOI:** 10.1177/0018726716661040

**Published:** 2016-08-25

**Authors:** Igor Pyrko, Viktor Dörfler, Colin Eden

**Affiliations:** University of Strathclyde, UK, igor.pyrko@strath.ac.uk; University of Strathclyde, UK, viktor.dorfler@strath.ac.uk; University of Strathclyde, UK, colin.eden@strath.ac.uk

**Keywords:** Communities of Practice, knowing, knowledge sharing, personal knowledge

## Abstract

In this article, we develop the founding elements of the concept of Communities of Practice by elaborating on the learning processes happening at the heart of such communities. In particular, we provide a consistent perspective on the notions of knowledge, knowing and knowledge sharing that is compatible with the essence of this concept – that learning entails an investment of identity and a social formation of a person. We do so by drawing richly from the work of Michael Polanyi and his conception of personal knowledge, and thereby we clarify the scope of Communities of Practice and offer a number of new insights into how to make such social structures perform well in professional settings. The conceptual discussion is substantiated by findings of a qualitative empirical study in the UK National Health Service. As a result, the process of ‘thinking together’ is conceptualized as a key part of meaningful Communities of Practice where people mutually guide each other through their understandings of the same problems in their area of mutual interest, and this way indirectly share tacit knowledge. The collaborative learning process of ‘thinking together’, we argue, is what essentially brings Communities of Practice to life and not the other way round.

## Introduction

The idea of Communities of Practice (CoPs) has been around for 25 years, and it has found its way into people’s professional and everyday language (Wenger, 2010). Put simply, CoPs refer to groups of people who genuinely care about the same real-life problems or hot topics, and who on that basis interact regularly to learn together and from each other (Wenger et al., 2002). However, operationalization of CoPs in organizational settings has proved challenging (Addicott et al., 2006; Swan et al., 2002; Waring and Currie, 2009).

This article aims to improve the clarity of the CoP concept by refining the explanation of why mutual engagement is an essential element of these social structures, and what that means. We introduce a trans-personal knowing process of *thinking together*, and we argue that without thinking together CoPs cannot exist. Thinking together is conceptually based on Polanyi’s (1962a) idea of *indwelling*: when peoples’ indwelling is interlocked on the same cue, they can guide each other through their understanding of a mutually recognized real-life problem, and in this way they indirectly ‘share’ tacit knowledge. Thus, thinking together allows for developing and sustaining an invigorating social practice over time. We synthesize the existing literature to construct an argument that CoPs come to life from peoples’ trans-personal processes of thinking together, and we substantiate the argument through the use of two empirical case studies.

The attempts to purposefully design CoPs face a critique for losing sight of the original emphasis placed on learning entailing an investment of identity in the social context, as well as losing sight of the spontaneous nature of CoPs (Amin and Roberts, 2008; Gherardi et al., 1998; Lave, 2008). As observed by Waring et al. (2013), some attempted to ‘set up’ CoPs in order to obtain knowledge as an output, which is reflected in the interventions where ‘CoPs-to-be’ were expected to implement certain pre-specified strategies based on ‘evidence’ (Anderson-Carpenter et al., 2014; Evans et al., 2014; Sabah and Cook-Craig, 2010; Tolson et al., 2008).

We demonstrate a similar skepticism towards the instrumental use of the CoP idea, which is not to say that CoPs cannot be intentionally cultivated – indeed success stories do exist, as illustrated by Saint-Onge and Wallace (2003). We agree with authors who view CoPs and knowledge as a process rather than an entity that can be simply ‘set up’ (Addicott et al., 2006; Corradi et al., 2010; Gherardi et al., 1998; Iverson and McPhee, 2002; Nicolini and Meznar, 1995). In order to better understand what CoPs are and how they can be cultivated in organizations, it is important to learn more about the learning processes that happen ‘in practice’ and that lead to CoP development (as seen in Gherardi et al., 1998; Handley et al., 2006; Iverson and McPhee, 2008; Kuhn and Jackson, 2008).

Drawing on these debates, in this article we seek to advance further the learning process view of CoPs. The structure of this article is as follows. First, the literature about CoPs is synthesized within the context of Michael Polanyi’s conception of *personal knowledge*, thus offering a consistent perspective on knowledge and knowing with the CoP concept. As a result, an argument is developed that *CoPs come to life from the trans-personal process of thinking together*, rather than, for instance, a community being ‘set up’ first. This argument is subsequently substantiated through two empirical studies in the context of two case studies set in the National Health Service (NHS). In the third section, we introduce a qualitative causal mapping approach in order to analyze the rich data collected through a series of semi-structured interviews. Subsequently, the findings are discussed in the light of the idea of thinking together, and organized around a number of propositions. These propositions are tentative and their main purpose is to help present the contribution of this article with regards to the nature of knowledge and knowing within the CoP concept.

## Conceptualizing ‘thinking together’

In this section, the idea of thinking together is conceptualized by drawing on the existing literature that addresses learning processes in CoPs. Initially, we discuss the role of knowledge and knowing as the way in which learning is portrayed in CoPs, although we note that knowledge, as a technical term, was missing from the original conceptualization of CoPs. We go on to acknowledge that later developments of the CoP concept make a distinction between knowledge and knowing in practice. Building on this discussion we explain that Polanyi’s idea of indwelling can be used to enrich the current understanding of knowledge and knowing in CoPs. Finally, we argue that based on the recognition that indwelling can be shared in practice when individuals interlock their indwelling on the same problem, thinking together is introduced as a trans-personal knowing process through which tacit knowledge is ‘shared’ indirectly and that essentially ‘brings CoPs to life’. The conceptual development introduced in this section will be substantiated through our empirical study in the next section.

### Knowledge and knowing in Communities of Practice

In CoPs, learning is portrayed as a social formation of a person rather than as only the acquisition of knowledge. Learning entails change in one’s identity, as well as the (re-)negotiation of meaning of experience. In the original formulation of CoPs the main focus is on the person becoming more competent in the context of idiosyncratic practice (Lave and Wenger, 1991). The formulation of CoPs was founded within a postmodern framework that tends to be skeptical about the notion of knowledge (as a term), associating it with appointed (or self-declared) experts who ‘monopolize’ the possession and creation of knowledge as their source of power. This explains why knowledge is silent in CoPs, being approximated with the concepts of learning, meaning and identity.

*Practice* is considered as ‘a set frameworks, ideas, tools, information, styles, language, stories, and documents’ (Wenger et al., 2002: 29). According to Wenger (1998), CoP members’ negotiation of meanings in practice leads to the development of three structural elements of CoPs: mutual engagement (how and what people do together as part of practice), joint enterprise (a set of problems and topics that they care about), and shared repertoire (the concepts and artifacts that they create). In CoPs, ‘belonging is enacted through the mutual engagement, sharing of repertoires, and negotiation of the joint enterprise(s)’ (Iverson, 2011: 43), and for an individual it may take different forms across different communities, ranging from full participation (‘leading the practice’ by the core group) to more peripheral or occasional participation (Handley et al., 2006; Wenger et al., 2002). Thus, being a member of a CoP is not necessarily something that people are aware of. However, they do still experience a sense of togetherness when, often owing to facing similar real-life problems, and not necessarily because of liking each other, they organize themselves around negotiating a practice that they all share and identify with (Wenger, 1998).

Furthermore, Iverson and McPhee (2008) applied mutual engagement, joint enterprise and shared repertoire in their ethnographic study of volunteering groups, which was helpful in identifying the specific characteristics and differences between CoPs based on how people interact ‘in practice’ rather than based on the labels that may be externally attributed to ‘possible CoPs’. What is particularly relevant to our discussion is that their research provided empirical evidence that CoPs cannot be ‘set up’ as formal teams, and that to better understand CoPs it is important to pay attention to the nuances of the lived practice.

Thus, the work of Lave and Wenger, as well as other early contributions to the CoP concept (Brown and Duguid, 1991; Orr, 1996), paved the way for the current popularity of the studies of knowing-in-practice (Nicolini, 2011; Orlikowski, 2002; Rennstam and Ashcraft, 2014), which was labelled as ‘the quiet revolution’ (Easterby-Smith et al., 2000). In the spirit of this approach, to put it simply, knowledge is potentiality to act, while knowing is using what one knows in practice. Following this perspective, knowledge ‘sticks to the practice’ in the sense that the potential to act is developed in the social context, but it also ‘leaks through the practice’ when practitioners from different contexts learn from each other as they try to address similar real-life problems (Brown and Duguid, 2001, 2002).

### Personal knowledge and indwelling

The foundation of reported research on personal knowledge and indwelling is that the understanding of knowledge and knowing in practice can be refined further by drawing more strongly on the contributions of Michael Polanyi who introduced a sophisticated conceptual model of human knowledge. Polanyi’s conception of personal knowledge offers a coherent view on knowledge and knowing that, importantly, is compatible with the essence of the CoP concept with its roots in identity. While Polanyi advanced considerably what is known about knowledge and knowing in the contemporary literature, there still remains much opportunity for building on his work. As Tsoukas and Vladimirou (2001: 975) write: ‘. . . no self-respecting researchers have so far failed to acknowledge their debt to Polanyi . . . [even though] Polanyi’s work, for the most part, has not been really engaged with’.

In order to unpack the role of indwelling in CoPs, it is important to understand Polanyi’s conception of personal knowledge. Central to Polanyi’s view of personal knowledge is the idea of the tacit component that is a necessary ingredient of all knowledge. ‘Personal’ implies that knowledge, in its richest form, can only exist within individuals and that it is necessarily grounded in the tacit dimension that people cannot easily say, as in Polanyi’s (1966b: 4) popular assertion that ‘we can know more than we can tell’. In other words, the tacit dimension can be thought of as the bottom of an iceberg that stands for the major part of what people know and that underpins everything that people know, and hence ‘a wholly explicit knowledge is unthinkable’ (Polanyi, 1966a: 7).

Thus, the tacit dimension to knowledge warrants that the personal coefficient is present in all knowing. Knowledge is developed through *indwelling*, which is an aspect of the knowing processes that accounts for learning (Polanyi, 1962a, 1966b). The process of indwelling captures the relationship of a knower’s body with the external world that they learn about as the *experience of everyday life*. From this perspective, a knower’s body includes rather than excludes the mind, and therefore indwelling applies to the development of both physical (e.g. sports) and intellectual knowledge (e.g. mathematics) – often at the same time. Peoples’ bodies, and thus their knowledge, is an instrument in relation to which they attribute meanings to the objects around them: ‘it is by making an intelligent use of our body that we feel it to be our body, and not a thing outside’ (Polanyi, 1966b: 16). Hence, people rely on their bodies, and so on their personal knowledge, while they attend to a focal point of attention in any given moment, as when surgeons dwell in their medical knowledge to perform a surgery using surgical tools, or pianists dwell in their musical knowledge to deliver a concert on a piano (see also Dörfler and Ackermann, 2012; Tsoukas, 2005).

So to a greater extent people dwell in a knowledge area, the more their bodies fuse with that knowledge area, thus their knowledge area becomes part of their extended identity. In general, such understanding of indwelling provides a considerable explanatory power; where, for example, it is possible to make sense of how Formula 1 drivers can legitimately claim that they feel the car as their body or mathematicians feel united with their equations. Specifically in the area of CoPs this understanding of indwelling offers more substance to the note of investing one’s identity in practice (Polanyi, 1962a, 1962b, 1966a, 1966b). Indwelling itself can also be shared but this requires putting trust in another person, as stated by Polanyi (1966b: 61):

In order to share this indwelling, the pupil must presume that a teaching which appears meaningless to start with has in fact a meaning which can be discovered by hitting on the same kind of indwelling as the teacher is practicing.

Although Polanyi mentions that indwelling can be shared, he does not elaborate on this aspect of the concept, as his focus is on personal knowledge, and shared indwelling is trans-personal. However, in CoPs the trans-personal dimension is essential, and thus we bring the idea of shared indwelling into the CoP concept. The notion of shared indwelling illustrates that people with different personal knowledge, but who manage to find ways to meaningfully attend to the same problems, can indirectly share their tacit knowledge by extending their identities into the same knowledge area. Indirectly sharing tacit knowledge in this sense means that each individual engaged in the trans-personal process of shared indwelling will (re)develop their tacit knowledge based on the experience of mutual performance in the shared lived practice. As they attend from their bodies to the problem, their shared indwelling becomes *interlocked* in the fleeting moment during which the extended identities of the participants overlap. Therefore, *interlocked indwelling*, as a trans-personal knowing process, can help understand forms of learning partnerships better.

We use ‘interlocked indwelling’ as a transitory concept, the role of which is to help us understand the essential knowing process at the heart of the CoP concept that we call *thinking together*.

### Developing Communities of Practice by ‘thinking together’

Having discussed the notions of tacit component and shared indwelling, we now use these ideas to develop the concept of *thinking together*. We expect that the emphasis on the process of thinking together may help to gain a better understanding of the nature of CoPs and their fundamental learning processes, which are of high relevance to anyone interested in operationalizing the CoP concept. By developing a better understanding of thinking together we hope to provide practitioners with a useful point of focus for fostering such communities in organizational settings. In the subsequent argument, we develop the concept of thinking together in three steps.

#### Thinking together entails interlocked indwelling

In the way indwelling is described by Polanyi. Hence, it is a trans-personal process through which people intensively learn together and from each other in practice, and in this way they become more competent practitioners. However, while indwelling explains how the deep mutual learning takes place, thinking together additionally brings indwelling into the CoP concept by placing an emphasis on the possibility of developing learning partnerships and a sense of community. Such learning partnerships can be achieved through mutual identification when individuals’ indwelling is interlocked: people engaged in thinking together guide one another through their understanding of the same problem. However, this understanding relates not only to technical, practical or theoretical knowledge (the main focus in indwelling), but also to the understanding of the (historical) relationships and communities that are relevant to the given practice. Thus, thinking together is inclusive of interlocked indwelling, but interlocked indwelling is not necessarily inclusive of thinking together. This, in turn, refines and elaborates McDermott’s description of knowledge sharing as thinking together:

Sharing knowledge is an act of knowing who will use it and for what purpose. This often involves mutually discovering which insights from the past are relevant in the present. *To share tacit knowledge is to think together*. (McDermott, 2000: 20; emphasis added)

Sharing knowledge involves guiding someone through our thinking or using our insights to help them see their own situation better. To do this we need to know something about those who will use our insights, the problems they are trying to solve, the level of detail they need, maybe even the style of thinking they use. (McDermott, 1999: 107–108)

#### Understanding thinking together as a form of sharing tacit knowledge under non-routine problematic circumstances

This addresses the need for using more refined language to talk about knowledge sharing (He et al., 2014; Konstantinou and Fincham, 2011; Sankowska and Söderlund, 2015; Wang and Noe, 2010). To share tacit knowledge through thinking together is more demanding that just a ‘quick question’ where there is ‘no obligation to delve into the matter until an answer could be found’ (Pentland, 1992: 537). It is more about situations where ‘people first understand the problem as experienced by the seeker and then shape their knowledge to the problem at hand’ (Cross et al., 2001: 105). As the idea of indwelling does not differentiate between body and mind, thinking together avoids the dualism between thinking and doing together, which would otherwise be incompatible with the conceptualization of CoPs (Wenger, 1998).

Furthermore, since it is reasonable to expect that opportunities for thinking together happen under non-routine problematic circumstances, thinking together can be related to Kuhn and Jackson’s (2008) *Framework of Knowledge Accomplishing.* According to this framework, knowledge is accomplished in practice through acts of knowing that range from more routine learning interactions where the provision of abstract information may suffice (knowledge deployment as information transmission/request) to more engaged mutual forms of knowing under non-routine problematic circumstances (knowledge development as instruction and improvisation). As thinking together can be safely positioned as ‘knowledge development’ in Kuhn and Jackson’s framework, this way it can be usefully contrasted against less intensive forms of learning.

#### Viewing thinking together as being necessary for Communities of Practice to thrive

This helps us to understand why mutual engagement of community members is required (Iverson and McPhee, 2008). Simply deploying knowledge in the form of casual information exchange rather than mutually engaging in more intensive knowledge development (Kuhn and Jackson, 2008) cannot sustain a thriving practice (Wenger et al., 2002). It calls for a view of knowledge sharing where knowledge is not transferred in a literal sense like an object, but it is re-recreated by knowers during those very acts of knowing (Bechky, 2003; Velencei et al., 2009; Von Krogh, 2011). At a conceptual level, the trans-personal process of thinking together is necessary for CoPs to thrive. This perspective is now explored further and substantiated through an empirical study.

## A study of Communities of Practice in the National Health Service Scotland

In this section, we use two case studies to both substantiate and illustrate the power of the concept of thinking together using the above two features of thinking together: (i) *interlocked indwelling*, and (ii) *sharing tacit knowledge*. And, in addition, provide support to the conclusion of the last section that *thinking together is necessary for CoPs to thrive*.

### Research design

The empirical study of CoPs was conducted in the NHS Scotland. We present cases from two different areas of NHS Scotland, namely: dementia and sepsis. The first case study describes a struggling CoP ‘to be’ while the second describes a thriving one; this contrast made it easier to observe salient characteristics, and it emphasizes the points we make. The empirical study had a qualitative character and it comprised of 29 semi-structured interviews or loose conversations with an average length of one hour each, and they took place in various hospitals across Scotland. The managers in the NHS Education for Scotland helped arrange the interviews with practitioners who expressed interest in the topics relevant to the study, and so a mix of purposive and snowball sampling was used (Biernacki and Waldorf, 1981; Teddlie and Yu, 2007). The practitioners were from CoPs at different stages of maturity and they were all healthcare practitioners (rather than patients, caretakers, etc.). The topics discussed covered social learning, their experience of CoPs, and the learning culture at their immediate workplace, with additional discussion on how each of these translated into better performance. While many participants had been aware of the concept of CoP, technical terms (including CoPs, thinking together, knowledge sharing) were presented and clarified during each interview when appropriate.

Following March et al. (1991) we attempted to ‘learn richly’ from this sample of practitioners by paying attention to the specific context of the particular CoPs, looking at the multiple aspects of the interview material, and thinking reflexively about alternative interpretations. The gathered data were rich and messy, and hence a method of analysis was used that could help to structure the data, make sense of it, and communicate the research results in a meaningful way, while not losing too much of its complexity. Our way of achieving this was through applying a causal mapping method in the analysis of data.

### Causal mapping

Causal mapping was used because it was able to respond to the demands of idiographic data (the interviewing deliberately encouraging open responses). Causal mapping is a formal technique where a person’s thinking about a problem is modelled using directed graphs. The structure of causal maps emerges from an analysis of the interview material by identifying possible causal relationships of concepts represented by short phrases (quotes) that are linked by unidirectional arrows that represent expressed causality (Eden, 1992; Laukkanen, 1994). Various approaches to causal mapping have been refined over the years, including both quantitative and qualitative ‘styles’, but each approach is governed by a set of guidelines that need to be followed for the resulting maps to be amenable to formal analysis (Bryson et al., 2004). Causal mapping is well suited for structuring, coding and making sense of rich, idiographic qualitative data from studies concerned with the explorations of social practice, as it was the case in this research. Causal maps of this type represents action-oriented statements connected by causal links signifying beliefs of the interviewee about how their world works. In this study, the maps were developed, represented and analyzed using a dedicated causal mapping software (Decision Explorer^1^). All the maps, constructed for each interviewee, were based on the audio recordings of the individual interview, and those separate maps were subsequently merged where the meanings of statements appeared similar. This process allowed us to immerse ourselves in the recorded conversation, and to pay attention to non-verbal cues (such as the tone of voice). Thus, the process of mapping was as important as the final map because it ‘forced’ careful listening by the researchers.

The final merged map comprised of 1869 statements connected in a network of causal links. Decision Explorer (software that allows both visualization of parts of the map and analysis) was next used to identify possibly interesting patterns in the network by using a range of analytical functions: domain analysis (direct in/out links for each statement), centrality analysis (multi-layered domain), and identifying presumed vicious and virtuous cycles. These analyses identified patterns that were copied into NVivo where the fragments of interview transcripts were uploaded. Consequently, two models of the data emerged that mirrored each other’s structure: a model in Decision Explorer and a model in NVivo. Using both models it was possible to jump quickly between the fragments of causal maps, the analysis in Decision Explorer, and the corresponding parts of the empirical material represented in NVivo.

In the next section, we discuss the findings from the two empirical case studies.

## Findings from the empirical study

Although the findings in this section are organized according to the two empirical cases, it is important to note that they were not obtained through a tidy linear process, but rather through a highly iterative one, actively switching between the two cases, and between the Decision Explorer and NVivo models, making the analysis clearer and more rigorous.

### Bringing dementia professionals out from isolation

The first case took place in the topical domain of dementia. In the UK, an important role in helping patients with dementia is performed by Allied Health Professionals (AHPs), a group comprising a number of professions specializing in supporting people, including dementia patients, through different types of therapy. AHPs include, among others, occupational therapists, speech and language therapists, art therapists and dieticians. These different professions did not naturally have mutual access to each other’s knowledge because of working in different locations and working with patients at different stages of their disease. A group of AHP leaders wanted to make a difference: they decided to bring dementia professionals out of isolation.

The AHP leaders believed that it would be beneficial to expose the dementia practitioners to each other’s practices and so reveal their otherwise inaccessible tacit knowledge. The proposal was intended to prevent ‘reinventing the wheel’ and allow for arriving at ways of doing things that seemed to work best for everyone, and contribute to seeing dementia from a more holistic perspective – as a journey comprising of different stages that all needed to be understood and looked after. In other words, the AHP leaders aimed for *engaging* practitioners in a shared practice; a description that seems to be a perfect fit with the CoP concept. The AHP leaders decided to ‘set up’ a CoP.

As a first step, the AHP leaders prepared a charter outlining what they expected from the CoP, what the benefits were likely to be, and also the required code of conduct for the future CoP members. Subsequently a discussion forum labelled ‘Community of Practice’ was designed, hosted by the health services’ library, fully open to the public with the expectation that in time it might reach a wider audience. As one of the AHP leaders later commented, this initial step to ‘set up’ the CoP had seemed relatively straightforward. Two of the AHP leaders became dedicated administrators of the discussion forum, their role involved uploading and organizing the content and monitoring the user activity. Moreover, to increase the recognition of CoP, the AHP leaders were promoting the discussion forum in informal conversations and in their email signatures. They also started to use the discussion forum to publish a quarterly newsletter about dementia that was based on the stories received from practitioners across Scotland about their day-to-day work. With time the newsletter became a success in the sense that the AHP leaders were receiving positive comments and emails not only from within Scotland but also from other parts of the UK and even from other countries. The newsletter did serve some of the AHP leaders’ original goals as it was promoting the work of dementia professionals and it was exposing them to each other’s practices. The AHP leaders were receiving more contributions from enthusiastic practitioners than they could possibly include in a single edition of the newsletter. Meanwhile, the sole purpose of the CoP discussion forum seemed to be to serve as one of the delivery channels for the newsletter, but there was very little conversation happening on its pages. As noted by one of the AHP leaders: ‘We try to encourage discussion, and that has not gone well. We’ve had people put questions out, and no answers. And we don’t know whether or not people are then replying outside of the discussion pages. They might be’ (Dementia Consultant).

When interviewed, the practitioners who had signed up to the forum typically explained that they had not been posting comments in the forum because of lack of time:

I think that it’s about managing your time. And actually allocating time. You know, so now I’m thinking: I need to allocate myself some time every week to go on to that CoP and just say that is the hour that I’m going to go on and I’m going to do that. (Alzheimer Scotland Dementia Nurse Consultant)

However, our analysis of the interview data (using the two software packages) suggested that the reason why people would not use the CoP site was that it did not provide them with immediate value to *justify their time investment* (the construct most central in the causal map model). In addition, they identified that there had not been conversations already taking place that they could join or observe and the range of topics had been defined in too general terms making it difficult to relate to more specific problems that could be of particular interest. Users would have been prepared to find the time to use the discussion forum if it had attracted them with something they perceived as immediate value, such as: engaging discussions, new working relationships, ability to share their views, solutions to their problems, opportunities to see what others are doing, and some tools, documents or techniques that they could use in their work. This observation is illustrated by the following quote, which is indicative of similar views expressed by most of the interviewees:

I think sometimes [in] that face-to-face kind of communication that you can have with your team . . . you [can] say: ‘I’ve encountered this problem today, what shall I do about it?’ . . . It’s *immediate*, and you get your response immediately. [Whereas] sometimes within the CoP you might post up a query, a dilemma, and *there’s no* actual guarantee that anybody will respond to it. (Dementia Liaison Team)

**Figure 1.**
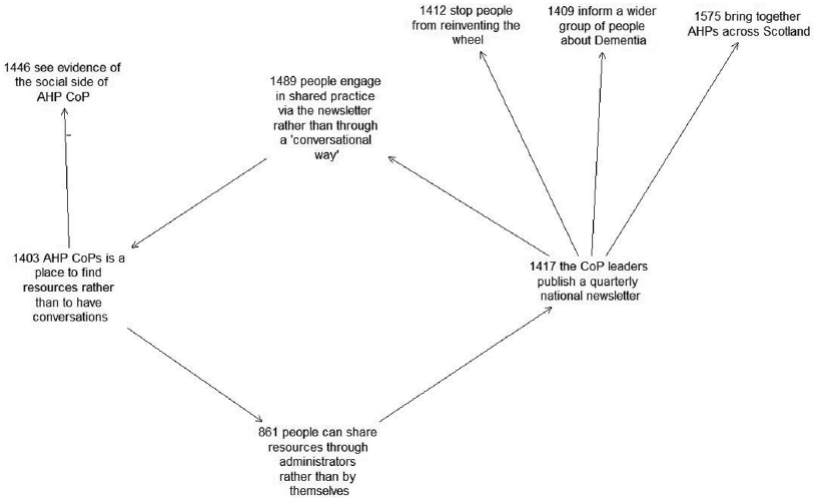
Dementia case. The causal arrows signify ‘may lead to’. The causal arrow with a minus sign signifies ‘may not lead to’. The numbers before statements signify the order in which they were added. Note that the picture represents a closed feedback loop of vicious nature – which means that it represents self-reinforcing negative circumstances.

Furthermore, our analysis of the causal map identified an interesting vicious cycle (Figure 1): people can only submit their resources through administrators, which leads to the consultants publishing the newsletter, which leads to people feeling encouraged to engage in the shared practice via the newsletter and not through direct conversation on the forum, which leads to the CoP website being a place for resources rather than a place for conversation, which then self-sustains the cycle.

This vicious cycle suggests that while the newsletter seems to have addressed some of the consultants’ goals, the emphasis on the newsletter was also a possible distraction from fostering direct conversations.

Gradually, it became apparent from the series of interviews that the AHP leaders confused the discussion forum labelled ‘Community of Practice’ with the actual CoP. While CoPs can and often do make use of various online tools, it is very important to draw a sharp line between the CoP and the tools it uses – the dementia story illustrates this well. In addition, although the discussion forum could have enabled interactivity that would at least qualify for marginal participation, this interactivity never took off as the forum became simply a delivery channel for the newsletter. The marginal success of the newsletter was even making less visible the fact that there was no CoP, that, using Kuhn and Jackson’s (2008) terminology, it was knowledge development that users needed, but what was happening on the discussion forum was merely knowledge deployment in the form of requesting and providing information. There was a community of practitioners that cared about the same real-life problems, and they *engaged* to some extent, but *did not mutually engage*. There were no opportunities for interlocked indwelling on things that the practitioners genuinely cared about, and so thinking together could not take place. The point of creating a discussion forum was merely a step towards cultivating a CoP, but definitely not the moment of actually establishing one.

After their initial participation in this research, the AHP leaders thought that thinking together was a useful way to talk in concrete terms about what it takes to foster a CoP. Feeding back the research results (outcome of the analysis of the maps and NVivo) enabled the AHP leaders to easily understand why there was not as much conversation happening on the CoP discussion forum as they had initially expected. While they were happy about what they had achieved with their newsletter, and as they wanted to continue working on it, they also decided to start a small informal learning group among themselves that they hoped might evolve into a broader community. Additionally, the NHS Education for Scotland, who were closely following our research with the dementia CoP, incorporated our research results in internal documents, invited us to give talks at their events, and we participated in various meetings around the CoP topic.

### Educating a hospital about sepsis

Our second case took place in the topical domain of sepsis. We concentrated on a team specializing in diagnosing and treating sepsis called the Critical Care Outreach Team (henceforth Outreach Team). The team and the hospital where they are based have been recognized both nationally and internationally for the quality of their work:

Analysis of the results has seen Borders General Hospital Intensive Care Unit record some of the lowest patient figures for out-of-hours admissions, length of stay, need for ventilation and need for renal replacement therapy in the country. On top of this the number of cardiac arrest calls at the hospital saw a remarkable reduction from 465 in 2000 [when the Critical Care Outreach Team was established] to 48 in 2013. (Healthcare Improvement Scotland, 2014)

At the foundation of the Outreach Team there was a need for improving the diagnosis and treatment of sepsis. It had been believed by its leader, a Clinical Nurse Specialist, that it was necessary to spread the active responsibility for diagnosing sepsis beyond intensive care (where very sick patients are treated). The reason for that need was that sepsis could occur anywhere in the hospital and therefore it was very important that as many practitioners as possible were confident about recognizing the symptoms early.

The Outreach Team comprises of five senior nurses who specialize in sepsis and who all have experience in intensive care. Not only is the team responsible for quickly responding to cases of sepsis in the hospital, but they also educate the staff in the wards about diagnosis and first response, provide them with supporting tools and systems, and help to improve their communication about sepsis. The importance of this education and communication was also evident from the analysis of the causal map. Moreover, analysis highlighted the facilitation of education about sepsis ‘on the job’ as the most significant (central) theme that enabled the whole hospital to develop its organizational ability to perform well in this area of strategic importance.

The range of regular actions of the team included: demonstrating to practitioners how to deal with sepsis ‘in practice’; mentoring junior doctors and junior nurses who are allowed to spend time with the team; organizing training courses about sepsis in the hospital, which are delivered by an interdisciplinary teaching team; designing objects that support interdisciplinary communication about sepsis such as small cards with key definitions, descriptions of symptoms and required actions, which are distributed among practitioners; and, convening interdisciplinary sepsis-related meetings where patient cases are discussed. As the leader of the Outreach Team commented:

We are a bridge between the intensive care and the ward areas. Historically, the intensive care was quite a secretive place. It was an *inner sanctum* that patients came to and then the nurses didn’t see them again until they come back out again. And there wasn’t any sort of joint working. And back where we started there was a nice term came out that ‘we should change and it should be *critical care without walls*’. Not physical walls, but metaphorical walls. And that was our starting point. There was lots and lots of different things. We got nurses who were in intensive care to go out and spend time in the wards to see what it looks like. And we got nurses from the wards to come spend time in intensive care. And that served a lot of useful things. People get to know each other. (The Outreach Team’s leader)

And then we saw an opportunity for another sort of learning: that if student doctors, student nurses, staff nurses came and spent time with us and see what we do, that would increase their learning. And to this day that’s growing and growing. So several student nurses as part of their training now they’re asked come and spend their time with us. And the student doctors as well. (The Outreach Team’s leader)

By the time we started our research there, the listed actions had become ingredients of the hospital’s sepsis-based practice, with a community of different types of practitioners organized around it. Practitioners from across the hospital identified themselves with sepsis because it could happen to their patients in the most unexpected moments. As a result, they genuinely cared about various real-life problems surrounding sepsis and they were willing to invest their time in learning more about it. Our interviews showed that owing to the Outreach Team’s work, people started ‘to come on board with sepsis’. In effect, the Outreach Team began to be seen as a leading group of a productive CoP (which had never been ‘set up’) with high impact, and with more peripheral members joining from various departments. As practitioners invested their identity in thinking together about what it meant to treat sepsis they not only acquired the useful facts and definitions but *became competent* in translating their learning into practice. The source of competence was the tacit knowledge that was being shared regularly among the CoP members. This sharing occurred partly in the ‘staff exchange’ between the wards and the intensive care and partly through the mutual engagement of the CoP members more generally.

I guess a lot of our success [of the Outreach Team] has been through education. A lot of nurses that see [the Outreach Team’s leader] in the ward, they learn something. [He] is a great teacher. (The Outreach Team’s member)

Our team is just part of a whole culture that’s changed. And maybe we have been a little bit of a catalyst in that change, or maybe instrumental. (The Outreach Team’s leader)

Although the leaders of the sepsis community had not been aware of the CoP concept, after we introduced them to this concept, and our use of the revelations from the causal map analysis, they agreed that it made sense to view themselves as a CoP. It is notable that even without any prior knowledge of the concept, the leaders cultivated a thriving CoP. The opportunities for interlocked indwelling on the same problems were given, and as genuinely interested practitioners they naturally engaged in building learning partnerships by thinking together. Furthermore, the members of the Outreach Team naturally emerged as core members through their mutual engagement. Owing to the core members’ outreaching activities, caring practitioners from various areas of the hospital started to engage in more or less intensive forms of participation, thus establishing the more peripheral layers of the emerging CoP. With regards to Kuhn and Jackson’s framework, the analysis of the interviews clearly showed that knowledge deployment was taking place in the form of mutual instruction and improvisation in the face of highly urgent, non-routine and problematic circumstances.

## Discussion of findings

In Section 2, we presented a conceptual discussion of the role of thinking together in CoPs. In Section 4, we described the findings from our case studies; in the light of these findings we now explore our conceptual claims about the role of thinking together in CoPs with respect to the empirical evidence. The contrast between the two cases, in the sense of one being only moderately successful and one being a thriving and high-performing CoP, gave us very promising research data. In the dementia case, the AHP leaders’ original goals and strategy seemed reasonable: they wanted to bring practitioners in their area out of isolation to enable them learn from each other’s experiences. They hoped it could improve professional practices and in effect achieve better care. Moreover, they wanted to follow the CoP approach because they had associated that concept with peoples’ active sharing of knowledge and with developing their competence together.

However, the main issue with the execution of their strategy was that they tried to ‘set up’ a CoP, focusing on the tools but neglecting the organic nature of the development of CoPs. They did provide an opportunity for mutual engagement by means of a discussion forum but did not provide opportunities for interlocked indwelling and thus did not prepare the avenues for thinking together. Furthermore, as the discussion forum was used as a distribution channel of the AHP newsletter, the discussion forum labelled CoP was perceived as a place for finding resources rather than for having conversations – knowledge deployment rather than knowledge development was taking place.

What the research showed was that the AHP leaders were lacking a group of people who could drive the learning. They could have helped that situation by identifying some more specific key problems and hot topics that were relevant to the organization and that the practitioners clearly cared about. They could have tried connecting people around problems and then supporting them or even join that core group if the others felt comfortable about their presence. Without thinking together about the same problems there was not enough mutual engagement that could sustain a shared practice and there was not enough value to attract less intensive forms of participation. Meanwhile, the codified stories submitted to the website administrators for the purposes of the newsletter (while valuable) did not substitute for it.

In the sepsis case, there was a thriving community because their members, as they indicated in the interviews, could see value in interacting regularly since they were holding a stake in similar problems or hot topics. Practitioners from various departments in the hospital were invited to learn together and from each other about sepsis. Instead of attempting to control what was happening in the wards, the team was taking the role of non-judgmental peer-mentors who supported other practitioners in developing their knowledge about sepsis in practice. Our research showed that deep tacit knowledge about sepsis was shared through active interlocked indwelling on real sepsis cases – for example, through regular peer mentoring of the nurses in the wards by the Outreach Team – which spread the knowledge of how to diagnose and treat sepsis beyond the intensive care that had originally been seen by practitioners as an ‘inner sanctum’. That gave birth to a community formed around the real-life need of recognizing sepsis early, which translated into a much better treatment of patients with sepsis within the hospital.

All of the analyses and illustrations highlight that it is important to look at community development as an emergent and continuous process where people think together regularly about real-life problems, in contrast with deliberately trying to ‘set up’ a CoP. As soon as thinking together at the heart of the community stops, it will quickly begin to lose its rhythm and vibrancy (or it may never come into life in first place). Thus, our empirical findings elaborate the previous findings in the literature and reconfirm mutual engagement and more specifically thinking together as a necessary component of CoPs (Addicott et al., 2006; Iverson and McPhee, 2002, 2008).

In order to bring focus to the contribution of this research, we set out below four tentative propositions that will act as a summary. Following the above discussion, our first proposition is therefore:

*Thinking together about real-life problems that people genuinely care about gives life to CoPs.*

Building on the first proposition, thinking together can also be related to Kuhn and Jackson’s (2008) ‘knowledge development’ as a form of knowledge accomplishment. In our sepsis case, the CoP gave opportunities for practitioners to learn how to deal with highly problematic situations involving the treatment of sepsis under high stress and urgency. The practitioners were clear that this could not be achieved merely by circulating documented guidelines, because practitioners needed to develop their tacit knowledge of treating sepsis that would allow them to help very ill patients ‘in practice’ at any moment (Orr, 1996). The deep, tacit knowledge was developed through regular thinking together within the context of the community, for example by inviting junior nurses and junior doctors to learn about sepsis from the Outreach Team ‘on the job’. Thus, our second proposition, which draws on Polanyi (1962a) and reconfirms the work of McDermott (1999, 2000), is:

*Thinking together, as a trans-personal knowing process, is a good way of sharing tacit knowledge. Knowledge is redeveloped rather than literally transferred from one person to another.*

Since thinking together is at the heart of CoPs, it helps to understand better the nature of CoP membership, as for example discussed by Handley et al. (2006). In the dementia example, at the beginning of the life of the CoP-to-be a group of AHP leaders agreed the objectives and the charter for their community. However, while their initial work in terms of establishing the community could certainly have been be useful, it did not mean that they were ready to regularly think together about problems among themselves or with other members of the community – especially that it might have required much more time and effort than they were willing or able to invest (also see Harvey et al., 2013). As a result, while the ‘CoP’ had individuals with officially assigned supporting roles, it lacked mutual engagement that could drive the learning, and in effect there was not enough existing thinking together to develop a thriving practice. As Wenger (1998) writes, practice is a history of learning in the social context, while learning is the driver of that history.

Developing that community perhaps could have been more successful, if it was not simply an attempt to ‘set up’ a CoP but fostering it through targeting people with some shared problems that they all cared about and who were willing to mutually engage in a social learning process. Whether a core group of members who regularly think together would evolve around that domain would have required more than just coordinating efforts on the part of the AHP leaders, that core group should naturally emerge from the organic nature of the CoP. Forming an official group of leaders could not be a substitute for such group – only a possible help. In other words, supporting and championing a CoP is not the same as actually being one of the members who regularly think together with respect to the joint enterprise of that community. Therefore, our third proposition is:

*The core group of a CoP is defined by thinking together and not just by having a role in supporting the community or by holding stake in its wellbeing.*

Throughout this study, we have seen CoPs be associated by different practitioners with informal groups, discussion clubs, social networking sites, or groups of interest. However, what makes a CoP is not its informality, openness for ideas, or flat structure. These can certainly be common and desirable ingredients of CoPs; yet CoPs can also be formal, official, or take the form of close-minded cliques that deny outsiders access to their learning (Wenger et al., 2002).

While CoPs do not have to be informal, they are fundamentally self-governed and they are driven by peoples’ regular thinking together. The scope of CoPs therefore includes those people who engage in thinking together regularly, and those individuals who have meaningful access to that thinking together. Access to the CoP entails at least elementary understanding of what is talked about and the ability to contribute to the shared practice (as in legitimate peripheral participation). Thus, a social space deserves to be called a CoP if it can be characterized by sustained thinking together that is enriched by less intensive forms of participation.

If the scope of CoPs is understood as the above, then one might think that such communities are rare if not extinct in today’s organizations. In a fast-paced business environment people do not have ‘the luxury of sustained engagement’. A competitive, vertically-structured, individualistic, or hierarchical space may indeed not necessarily be offering the most suitable conditions for developing sustainable learning partnerships (Harvey et al., 2013; Roberts, 2006).

For those who want to implement the CoP concept, some useful questions are: Does it make sense to look at that social structure as a CoP? Would it be worthwhile or rather counterproductive? The accepted indicators (Wenger, 1998) that a CoP exists (for example, quick setup of problems, overlapping descriptions of who belongs) can be helpful in answering these questions. One reason for introducing ‘thinking together’ to the CoP concept has been to collate all those different indicators into one point of focus making it easier for practitioners to judge by themselves.

The findings discussed in this article indicate that the value of the CoP concept can be very limited when at least its most basic conceptual frameworks are not explored. Cultivating CoPs is not about deciding to ‘set up a CoP’, but about making conscious efforts to learn more about one’s own learning and ways of improving it. This insight then confirms our conceptual findings based on the literature (Addicott et al., 2006; Corradi et al., 2010; Gherardi et al., 1998; Iverson and McPhee, 2002; Nicolini and Meznar, 1995; Wenger et al., 2002).

CoP development requires establishing a stronger link between the lived experience of what it means to learn socially with other people, and with the CoP concept that aims to shed light on the complexity and the richness of such partnerships (see Iverson and McPhee, 2008). A more intentional use of a well understood CoP concept could have helped to overcome the community challenges in the dementia case, and to potentially make more of the existing social learning in the sepsis case. As a result our fourth, and final proposition is:

*The scope of CoPs is delineated by sustained thinking together of the core members enriched by less intensive forms of participation of those who have meaningful access to that thinking together.*

## Concluding remarks

The idea of thinking together is as important from an academic point of view as it is from a practitioner standpoint. From an academic aspect, the notion of thinking together elaborates the very foundation of the CoP concept by explaining the learning processes happening at the core of such communities and assigns them a central role. At the same time, thinking together does not replace the existing models that describe learning in CoPs, such as Wenger’s (1998) three structural elements of CoPs (shared repertoire, mutual engagement and joint enterprise), but it helps to better understand them. The three structural elements are developed specifically because of thinking together taking place, and therefore at the conceptual level they can be used alongside thinking together, and so helping achieve a deeper understanding of the structural elements.

From conducting our two case studies, we have found that thinking together was the term the practitioners could make sense of when trying to conceptualize CoPs. Significantly, for both academics and practitioners, the process of thinking together defines both the core and the scope of a CoP, and it explains why CoPs can be cultivated but not managed, because thinking together cannot be simply imposed by managers who decided that they ‘want to have a CoP’. Consequently, practitioners who engage in CoP development are encouraged to focus on building avenues for regular thinking together about real-life problems that people genuinely care about. A focus on thinking together refines further the existing work on cultivating CoPs as for example outlined in the works of Wenger et al. (2002) and Saint-Onge and Wallace (2003). In addition, we see a promising future research direction about exploring the use of causal mapping to support the process of thinking together in a CoP.

Furthermore, with regards to thinking together, it is possible to improve the current understanding of knowledge and knowing in CoPs through adopting the concepts of interlocked indwelling and thinking together. In Kuhn and Jackson’s (2008) framework, thinking together can be associated with knowledge development under problematic circumstances, in contrast with routine, casual and well-structured exchanges of information that are insufficient for thriving practice. Although in this article we have focused on the knowledge sharing aspect, thinking together also includes knowledge creation that is consistent with Kuhn and Jackson’ framework. Future research might explore the knowledge creation role of thinking together, as well as the adoption of thinking together as a perspective for interpreting and comparing the nuances of the practices of different communities, and so, for example, build on the work of Iverson and McPhee (2008). Similar investigations, possibly of ethnographic design, could possibly lead to a rich portrayal of thinking together in CoPs, with different types or forms of thinking together happening at various stages of the CoP lifecycle.

Finally, thinking together clarifies the notion of knowledge sharing, which is very popular in the literature especially in the field of Knowledge Management, and which can be relevant to practitioners by placing an emphasis on the mutually engaged social learning processes as an essential source of CoPs. Thinking together offers a perspective on knowledge sharing that is compatible with the Polanyian epistemology. In the light of the concept of thinking together, an assumption that knowledge can be literally transferred from one person to another can be considered as naïve; instead thinking together stresses that tacit knowledge is shared only in the sense that it is redeveloped as people discover each other’s performances in practice and they learn together and from each other, rather than being acquired or replicated.

## Notes

1 Decision Explorer is causal mapping software available from www.banxia.com.
